# Synthesis and Comparative Study of Nanoparticles Derived from Bovine and Human Serum Albumins

**DOI:** 10.3390/polym12061301

**Published:** 2020-06-06

**Authors:** Yerkeblan Tazhbayev, Olzhas Mukashev, Meiram Burkeyev, Vladimir I. Lozinsky

**Affiliations:** 1 Chemical Materials Science and Nanochemistry Laboratory, Buketov Karaganda State University, Karaganda 100028, Kazakhstan; mukashevoe@gmail.com (O.M.); m_burkeev@mail.ru (M.B.); 2 A.N. Nesmeyanov Institute of Organoelement Compounds, Russian Academy of Sciences, Moscow 119991, Russia; loz@ineos.ac.ru

**Keywords:** biopolymeric nanoparticles, drug carriers, bovine albumin, antitumor agents

## Abstract

This study describes the preparation of nanoparticles derived from bovine serum albumin (BSA) in comparison with the formation of nanoparticles composed of human serum albumin (HSA), when the same preparation procedure was used in both cases. To obtain protein nanoparticles, the method of desolvation with ethanol was employed, followed by the stabilization with urea and cysteine. It was shown that, upon transition from HSA to BSA, the particles with smaller sizes and with a narrower polydispersity were formed. The possibility of the immobilization of the antitumor drug hydroxyurea in such protein nanoparticles by adsorption and inclusion methods has been shown. The drug release profile from the polymer matrix was established.

## 1. Introduction

One of the important tasks of modern medicine is the development of inert and safe drug carriers that would ensure the sustained release and specifically directed transport of biologically active substances toward the target organs. This is especially true for the anticancer drugs, because of their high toxicity and low selectivity to tumor cells. Serum albumin, often of bovine origin, is among the biopolymers used for the development of carriers for transport of anticancer drugs [1,2]. The albumin content in blood serum is more than 50% of the total proteins [3]. Thus, in view of prevalence, serum albumin is an inexpensive object for use as drug carriers (especially when, thanks to its easy availability, bovine albumin is used). It is also known that, in the blood, albumin binds many metabolites that are often toxic, such as bilirubin, fatty acids, etc [4]. Blood serum albumins also have an excellent ability to bind the drug molecules [5].

Another useful natural function of serum albumin in an organism is to maintain osmotic pressure in the blood vessels; therefore, the solutions of this protein are often used as a plasma substitute in the case of large volume of blood loss. Along with the above advantages of serum albumin, its attractiveness for use as a drug carrier is due to the presence of a large number of side amino, carboxyl, amide and other functional groups in its structure [6,7]. There are known examples of serum albumin application to prolong the action of drugs of protein origin such as “Albuferon” and “Levemir” [8,9]. Another example is the antitumor medicine “Abraxane”, in which serum albumin is used as a polymeric matrix of the drug carrier. This pharmaceutical formulation is recommended for the treatment of breast cancer [10,11,12,13].

Various synthetic approaches have been reported for the preparation of serum albumin-based nanoparticles that carried certain antitumor drugs. These particular approaches are dependent on the nature and properties of the biologically active anticancer substance incorporated in such particles [14,15,16]. One of such examples is the desolvation-induced preparation of nanoparticles derived from serum albumin, where glutaraldehyde (a rather toxic compound) was additionally implemented in order to stabilize the forming proteinaceous particles [16].

We previously described the possibility of stabilizing protein nanoparticles without the application of non-proteinaceous crosslinking agents, and due to the formation of the intermolecular disulfide bridges with the participation of own thiol groups of the human serum albumin macromolecules [17]. This effect was achieved by the sequential exposure of the nanoparticles to chaotropic agent, such as urea, and a low molecular weight thiol—cysteine. The aim of the present study was to establish possible differences in the formation of similar nanoparticles derived from two types of serum albumin, namely of human and bovine origin. In addition, physicochemical characteristics and stability of the respective nanogels have been examined. We also entrapped the antitumor drug, hydroxyurea, inside the prepared protein nanoparticles and studied its release in order to evaluate such formulations as potential drug-loaded nanocarriers.

## 2. Materials and Methods

### 2.1. Materials

Human serum albumin (lyophilized powder, 98%) (HSA), bovine serum albumin (lyophilized powder, 98%) (BSA), and L-cysteine (98.5%) were purchased from Sigma Aldrich (Saint Louis, MO, USA). Absolute ethanol was purchased from DosFarm (Almaty, Kazakhstan). Urea (99.5%) was purchased from HimPribor-SPb (Saint Petersburg, Russia). Hydroxyurea (hydroxycarbamide) was purchased from the Bristol-Myers Squibb Company (New York, NY, USA).

### 2.2. Preparation of Drug-Free Nanoparticles Derived from BSA and HSA

BSA- and HSA-derived nanoparticles (NPs) were produced by the desolvation method in accordance with a slightly modified procedure described elsewhere [17]. In brief, the pre-set amount of serum albumin powder (0.03, 0.06 or 0.09 g) was dissolved in 3 mL of Milli-Q water (pH ~7.4) by stirring at 200 rpm, avoiding clumping and frothing, at 23 °C for 10 min. The concentrations of these prepared protein solutions were 10, 20 and 30 mg/mL, respectively. Thereupon, 16 mL of ethanol was added to each protein solution at a rate of 1 mL/min, thus resulting in the turbid dispersion of albumin nanoparticles. Then, 0.5 mL of aqueous solution of urea (its concentration was either 5 or 10, or 20, or 40 mg/mL) was added followed by introduction of 3 mL of aqueous L-cysteine solution, in which the amino acid concentration was either 0.001, or 0.01, or 0.1, or 0.3, or 0.5 mg/mL. Every reaction mixture obtained in this way was stirred continuously for 2 h. The resultant suspension of nanoparticles was rinsed using three centrifugation steps (MiniSpi, Eppendorf, Hamburg, Germany) at 14,000 rpm for 15 min each to remove dissolved substances and ethanol from the mixture. The precipitated nanoparticles were re-dispersed in 10 mL of deionized water after each phase of centrifugation using an ultrasonic bathtub (Launch Tech, Shenzhen, China); the ultrasound period was 10 min.

### 2.3. Preparation of Proteinaceous Nanoparticles Loaded with Hydroxyurea

Two approaches were employed in order to prepare the serum albumin-based nanoparticles loaded with hydroxyurea: (i) adsorption and (ii) inclusion.

(i) The process of serum albumin-derived nanoparticles, loading with hydroxyurea (HU) through the adsorption, consisted of the synthesis of the drug-free nanoparticles (Section 2.2) and their subsequent loading with the drug. For this aim, the hydroxyurea was added to 22 mL of the nanoparticles’ aqueous suspension (HU concentration was either 2, or 4, or 6 mg/mL) in an amount of either 45 or 90, or 135 mg. The resulting suspensions were stirred at a constant rate of 200 rpm for 2 h. The final stage was the centrifugation of the suspensions in three cycles at 14,000 rpm to remove the unbound HU.
(ii) The protein nanoparticles have been fabricated essentially in the same way as described above in Section 2.2, except for after completion of the desolvating process, 3 mL of aqueous HU solution (2 mg/mL) was added to the nanoparticulate dispersion followed by the system treatment with urea and L-cystein mixture under the conditions analogous to those in Section 2.2.

### 2.4. Size Measurement, Fourier-Transform Infrared Spectroscopy, Zeta Potential Analysis, Surface Morphology and Thermal Analysis of Serum Albumin-Derived Nanoparticles

To determine the average size of nanoparticles, we used a device based on dynamic light scattering (DLS) (Zetasizer NanoZS90, Malvern Instruments Limited, Worcestershire, UK). The samples were dissolved in deionized water at 25 °C. The instrument was equipped with a 90° angle light-scattering unit. Zeta potential was determined by a zeta potential analyzer (Malvern Instruments, Worcestershire, UK) based on electrophoretic laser Doppler anemometry. A scanning electron microscope (MIRA 3LM TESCAN, Brno, Czech Republic, EU) was also used to determine sample sizes.

Infrared spectra were recorded using the FSM 1201 FTIR spectrometer (Infraspek Ltd., Saint-Petersburg, Russia). Each sample was prepared with the KBr, pressed pellets were prepared in ratio sample 1 mg: 200 mg KBr. The detection range was from 4000 to 400 cm^−1^. Spectral resolution was 1 cm^−1^ on the used device.

Simultaneous Thermal Analysis (Thermal gravimetric analysis (TGA) and differential scanning calorimetry (DSC)) were performed using a LABSYS evo STA 1600 °C (Caluire et Cuire, France, EU). The samples (n = 3) weighed between 1.0 and 2.0 mg were measured. The ready-made samples were gradually heated from room temperature to 500 °C with a heating rate of 10 °C/min. TGA and DSC tests were conducted in intervals of immediate, 24 h, and 5 days.

### 2.5. Yield and Drug Loading Efficiency of BSA(HSA)-NPs

Protein-NP yield was determined by UV spectrophotometry (Shimadzu UV-1800, Kyoto, Japan). The typical curve of a protein dissolved in a phosphate-salt buffer (PBS) was used as a standard reference. The protein absorbance level was recorded at 280 nm. The concentrations of standards in the range from 1 to 5 mg/mL of albumin were used to create the calibration curve (standard linear curve equation *y* = 0.0198*x* − 0.0003 (*R* value = 0.9973)). This formula was applied to the yield calculating.

Yield [%] = (mass of albumin in solution/initial mass of albumin used) × 100.(1)

Protein-HU-NPs were collected by centrifugation using Amicon filters (Cedarlane, Burlington, ON, Canada) with molecular weight cut-off (MWCO) 10,000 Da to find out the efficiency of hydroxyurea encapsulation in NP proteins. This made possible the nonencapsulated HU to be eluated in a tube for collection. The nonencapsulated HU concentration was found by the high-performance liquid chromatography (HPLC) method (Shimadzu LC-20 Prominence, Kyoto, Japan).

### 2.6. Determination of the In Vitro HU Release

For the analysis of unbound HU substance quantity, the standard curve of dependence of optical density on HU concentration was made [18], and the calibration curve performed standards at different concentrations. For this purpose, aqueous solutions were prepared with various concentrations of the drug (0.5–6 mg/L of hydroxyurea). The device detector was tuned to a wavelength of 214 nm. Mobile phase of acetonitrile-water (95:5) vol/vol, isocratic elution mode, volume flow rate 1.5 mL/min. An Agilent 300 Extend (Agilent Technologies, Tokyo, Japan) C-18 (sorbent graining 5 µm, 4.6 mm × 250 mm) column was used. The column is equipped with a ZorbaksGuard pre-column (4.6 mm × 12.5 mm). The operating temperature of the column was maintained at 40 °C. Quantitative calculation method—internal normalization by area. The device was configured for injection with a volume of 10 µL (loop injection). The time required for the test was 10 min. Symmetrical HU peaks were detected at a retention time of 6.2 min. The release period was recorded at intervals of 0, 2, 4, 8, 12, 18 and 24 h. The quantity of the drug that was absorbed by protein nanoparticles was counted by the following equation:

Encapsulation efficiency (EE%) = (concentration of HU encapsulated/starting concentration of HU used) × 100.(2)

Three initial drug concentrations were used to analyze the release of the drug from nanoparticles. Samples with a concentration of 2 mg/mL were labeled as HU-2, 4 mg/mL = HU-4 and 6 mg/mL = HU-6. The nanoparticles loaded with the drug (BSA-HU-NPs) for analysis were prepared in the following way, first dissolved in 5 mL of PBS and then kept at the temperature of 37 degrees using a magnetic stirrer at the speed of 200 rpm. The quantity of HU released into the environment was measured at an interval of 24 h and compared with a control sample containing NP-proteins without HU.

### 2.7. Statistical Analysis

All the experiments were conducted in triplicate and presented as mean values ± standard deviation (SD). Statistical significances were analyzed using the Student’s *t*-test Statistica 12 (TIBCO Software Inc., Palo Alto, CA, USA). The probability level of 0.05 was considered statistically significant.

## 3. Results and Discussion

### 3.1. Optimization of Nanoparticles Preparation

One of the known methods for the preparation of albumin nanoparticles is the desolvation procedure [19]. Its scheme includes the addition of a desolvating agent, most often ethanol, to an aqueous solution of albumin, and subsequent stabilization of the forming particulate matter with a cross-linking agent [20,21].

**Figure 1:**
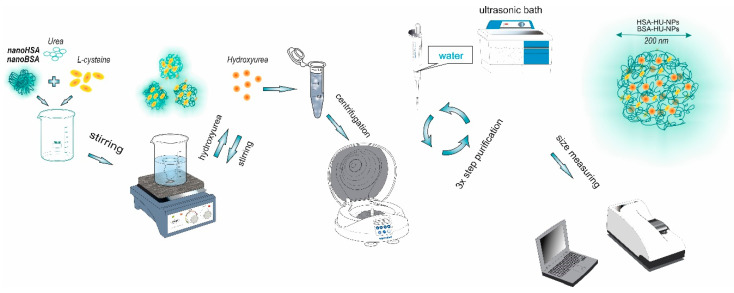
The schematic illustration of the method used to prepare HU-loaded protein nanoparticles.

Commonly, glutaraldehyde is used as a cross-linking agent [22,23,24,25,26,27,28]. Previously with the glutaraldehyde we prepared the albumin nanoparticles with a size of 182 ± 8 nm and a polydispersity of 0.14; then such particles have been loaded with various antitumor drugs [29]. However, as we described above, in this study an attempt was made to move away from the standard methodology and the cross-linking agent, i.e., glutaraldehyde, was replaced with a combination of urea and cysteine (Figure 1).

**Figure 2:**
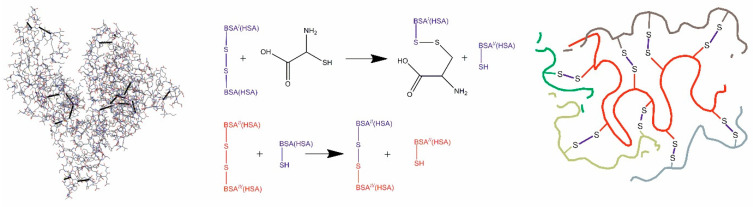
Cysteine interaction with BSA(HSA) (the left part is structural arrangement of double sulfide bonds in albumin, the right part is a schematic representation of bounded particles).

Urea is well known as an agent which causes protein denaturation thanks to weakening hydrogen bonding and hydrophobic interactions in protein molecules [30]. This is related to strong ability of the denaturant to form hydrogen bonds with polar functional groups present in biological macromolecules [31]. Thus, urea as a chemical substance affects the conformation of serum albumin, causing a partial unfolding of its globule with exposing the hydrophobic amino acid units to the surface. Next, cysteine enters the reaction and is capable of cleaving intramolecular disulfide bonds in protein macromolecules, which leads to the better unfolding of the polypeptide chains (Figure 2). As a consequence, because of the thiol-disulfide exchange reactions covalent intermolecular SS-crosslinks are formed [17,32,33,34]. In addition, a significant contribution to the stabilization of the spatial structure of polymer nanogels can be made by the hydrophobic interaction and, to a lesser extent, de novo formed hydrogen and ionic bonds.

**Figure 3:**
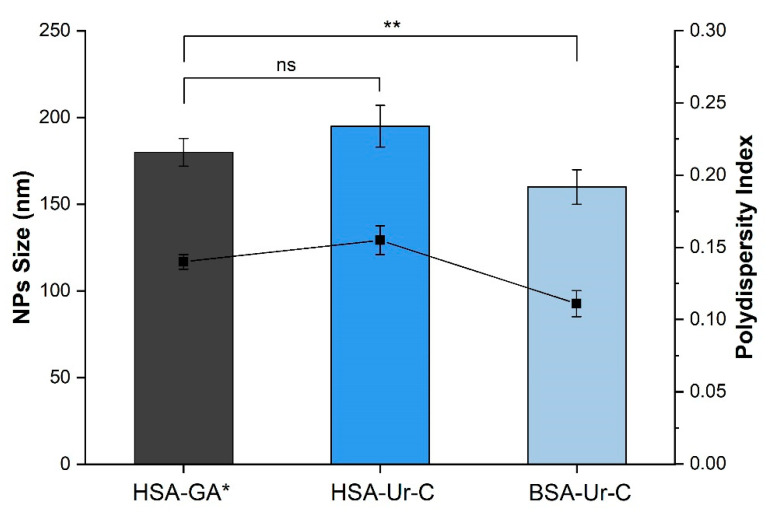
Effects of the type of serum albumin and cross-linking agent on the size and polydispersity index of NPs (the size values are depicted as columns, and the polydispersity index is shown as the solid line). * Albumin nanoparticles with glutaraldehyde (HSA-GA) were prepared according to the procedure described in [34]. Data are presented as mean ± SD (*n* = 3), ** *p* < 0.001; ns = no significant.

We have compared human and bovine serum albumin as the basis for nanoparticles. The results of the measurements of the size and polydispesity for NPs synthesized on the basis of HSA and BSA by desolvation in ethanol and then stabilized either with glutaraldehyde (GA) [1] are presented in Figure 3.

**Figure 4:**
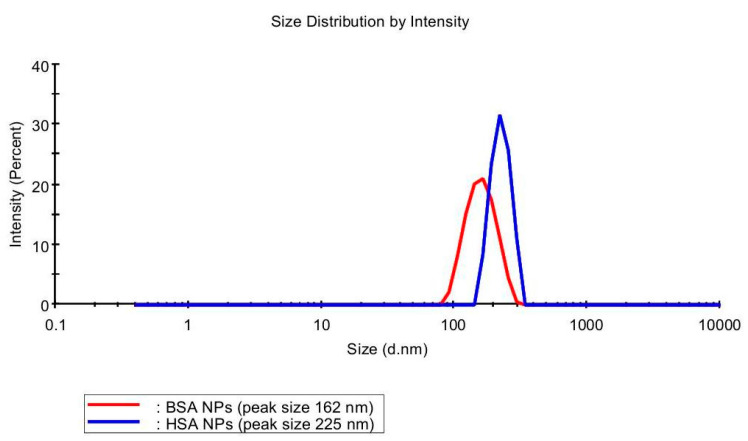
Exemplar size distributions of BSA and HSA nanoparticles measured by DLS.

It was found that replacement of glutaraldehyde for the urea/cysteine mixture makes it possible to obtain particles with a suitable size and dispersion index. As for the differences in the NPs derived from HSA and BSA in the albumin–urea–cysteine system, the latter case leads to the creation of smaller particles (particle size 162 ± 2 nm) and a more uniform size distribution (polydispersity index (PI) 0.11). The data obtained using DLS clearly demonstrate this (Figure 4).

Thus, the results presented above show the promise of using a mixture of natural agents, urea–cysteine, instead of glutaraldehyde for the synthesis of albumin NPs. In this case, no special external conditions are required, i.e., environmental parameters remain the same. However, as will be shown in the next chapter, the process of formation of NPs significantly depends on the concentration of urea and cysteine, as well as the origin of the protein. The average particle size for BSA-derived nanoparticles is slightly smaller 162 ± 5 nm. Thus, the formation of finer particles is preferable due to a more beneficial pharmacological profile, long-term kinetics in the circulatory system as well as delayed releasing at target sites [35]. The zeta potential was negative in both of these scenarios, indicating particle stability due to the forces of repulsion of the same surface charges. Taking into account the efficiency of HU encapsulation in NPs, its size and the Zeta potential, BSA-based samples were selected for testing the release and characteristics of the drug in vitro.

### 3.2. Loading of Serum Albumin Nanoparticles with Hydroxyurea

The above-described proteinaceous nanoparticles have been further used for the immobilization of the antitumor drug, hydroxyurea. This biologically active substance is available and inexpensive drug recommended for breast cancer treatment [35]. The important thing is that the structure of hydroxyurea does not contain any functional groups capable of forming chemical bonds with albumin molecules.

There are two most common approaches for immobilizing drugs into polymeric nanoparticles. The first method is the drug adsorption by the already prepared nanoparticles. The second method is known as the inclusion method, where the drug is injected into the reaction medium directly during the formation of nanoparticles. Both these variants are known to be used [36,37,38], and have their advantages and limitations.

In the present study, hydroxyurea was immobilized onto albumin nanoparticles by adsorption. The findings of the relevant experiments are presented in Table 1.

We previously described the effectiveness of HU encapsulation into HSA nanoparticles [17]. In Table 1 we summarize the physicochemical parameters of BSA–HU-NPs prepared at the optimal protein concentrations. The particle size increased as compared to the HU-free nanoparticles by about 20–30%, and in the case of HSA-derived NPs changed from 216 to 287 nm, for the BSA-derived NPs grew from 157 to 175 nm (Table 1). For the HSA-based specimens, the index of polydispersity changed insignificantly after HU absorption, whereas for the BSA–HU-NPs formed originating from the 0.2 g/mL feed solution PI values almost doubled (from 0.093 and 0.181), but in permissible limits. A high negative value of the zeta potential indicates charge stabilization of the studied nanoparticles, and a significant difference for HSA–HU-NPs and BSA–HU-NPs was observed.

Another promising approach for drug loading in the protein NPs is the inclusion method. In this method, drug immobilization is performed directly during the formation of albumin nanoparticles. The protein concentration was 2 mg/mL for this method, which should improve the efficiency of drug integration into nanoparticles. The results are presented in Table 2.

There was some difference in the size of NPs derived from HSA and BSA encapsulation. For both systems, a size distribution profile is monomodal (Figure 4) with the PI values for NPs based on BSA and HSA being 0.242 and 0.284, respectively. This parameter is a problem in obtaining nanoparticles because the size influences the biological and chemical properties of NPs [39,40]. Analogously to the case of HU loading in NPs with the absorption method (Table 1), upon using the inclusion approach the loading efficiency was higher for the BSA-derived particles (78%) in comparison to the HSA-derived drug carrier (69%) (Table 2).

### 3.3. Physicochemical Characteristics of BSA-NPs with Immobilized Hydroxyurea

Before the measurements, all samples were diluted with a 1-mM PB buffer. A 20 µL solution was dropped onto a glass flask, graduated and able to withstand high temperatures for continuous drying. Drying was carried out by gradual heating and a stream of inert gas. Sample preparation was carried out in the «SorbiPrep» complex.

**Figure 5:**
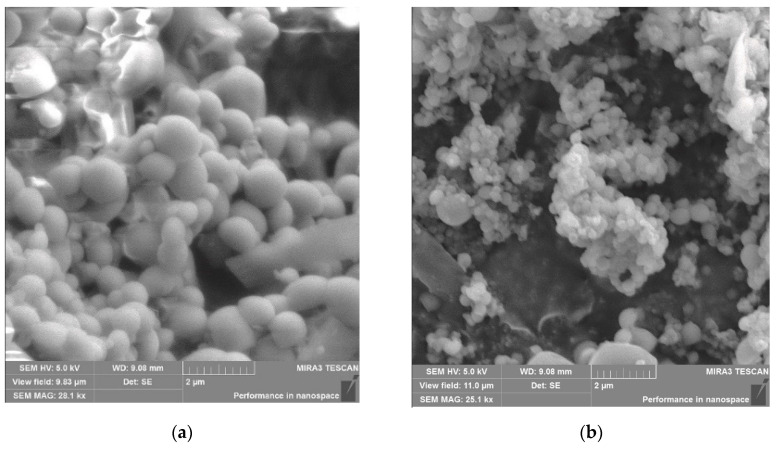
(**a**) Scanning electron microscopy (SEM) images of BSA-NPs (160 ± 5 nm) (scale = 2 µm, 5 kV). (**b**) SEM image of BSA-HU-NPs (180 ± 7 nm). Data are presented as mean ± SD (*n* = 3).

Morphological analysis of BSA-NPs and BSA–HU-NPs samples was performed using SEM, and the obtained images are shown in Figure 5a,b. The obtained nanoparticles of both types were spherical in shape and an average size of less than 250 nm (160 ± 5 and 180 ± 7 nm, respectively).

Microscopic data indicate that BSA nanoparticles are predominantly round in shape and not prone to aggregation (Figure 5a). When a drug substance is immobilized, the shape of the particles changes somewhat, they deviate more and more from the correct shape, but retain roundness. The size of BSA–HU NPs is 20–30% higher compared to NPs without drug.

**Figure 6:**
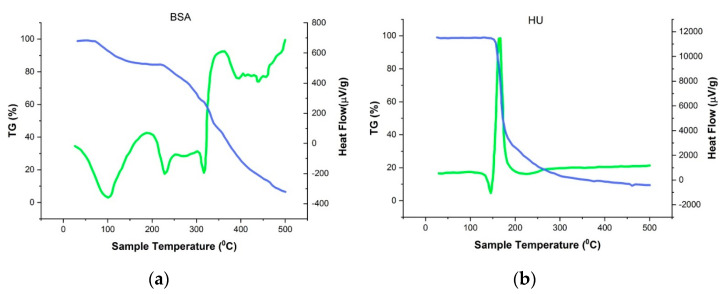

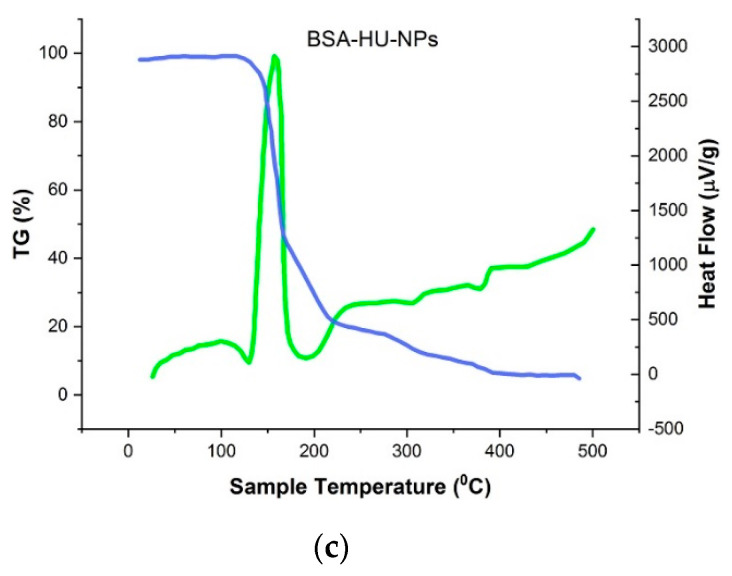
Thermogravimetric (blue) and differential scanning calorimetry (green). (**a**) BSA; (**b**) HU; (**c**) BSA-HU-NPs.

In order to make sure that HU is part of the albumin nanoparticle complex, thermogravimetric and DSC analyses of the individual components of the system and the obtained NPs were performed (Figure 6). From the graphs presented in Figure 6b, it is seen that the peak recorded at 144.6 °C corresponds to the endothermic peak of hydroxyurea. Figure 6b shows the phase transition of hydroxyurea. Detected change of hydroxyurea enthalpy shows that the process is involved with no mass loss (temperature close to melting point). At further increase in temperature there is an exothermic peak at 167.8 °C, in the area between 155–180 °C; mass loss in this case corresponds to 53%, which may be associated with the degradation of the drug [41,42]. The recorded endothermic value of BSA-NPs peak at 95.8 °C (Figure 6c), which most likely matches their melting period. However, in comparison with pure BSA TGA curves (Figure 6a) a substantial movement to the area of higher temperatures was observed [43]. Perhaps this is due to the huge structured albumin in NPs. In addition, when heating the BSA-NPs sample on the DSC curve area at 229.5 °C, there is another endothermic peak, which fully matched with pure albumin not being structured into nanoparticles. The following peak on the DSC curve represents the decomposition of protein NPs at 313–351 °C with a more than 50% mass loss.

**Figure 7:**
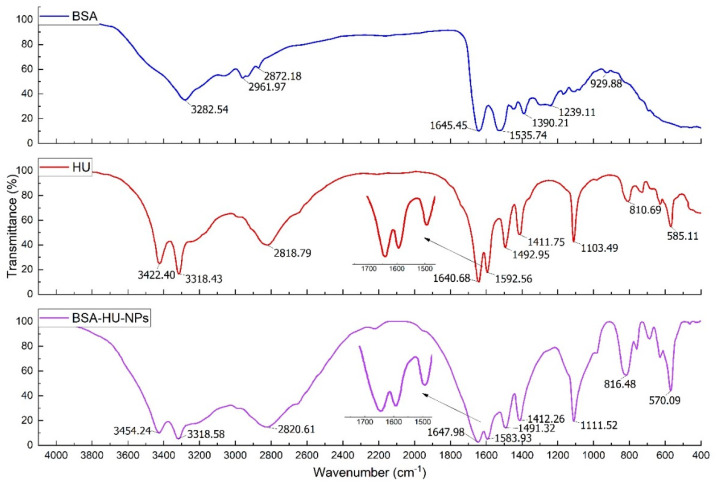
FTIR-Spectra of BSA, antitumor drug hydroxyurea (HU) and complex BSA-NPs with hydroxyurea.

FTIR spectra of pure BSA and immobilized HU nanoparticles were obtained (Figure 7). The presented spectra consist of the bands of original albumin detected at 3284 cm^−1^ (A—amide, linked to N-H), the next pointed peak at 2960 cm^−1^ (B—amide, bound to free ion), amide II recorded peak at 1535 cm^−1^ related to C-N stretching and N-H bending movements; the amide I peak at 1642 cm^−1^ corresponds to the C-O bond; detected CH_2_ groups are located at 1393 cm^−1^, and the amide III at ~1245 cm^−1^ linked to the C-N group stretching and N-H bending. The main area for the study of protein secondary structure is amide I [44]. The peak shift from 1642 to 1650 cm^−1^ after interaction with the drug is visible from the spectrum. It should also be noted that the observed changes in the intensity of the bands, namely, B—amide and amide III, indirectly show the interaction of albumin molecules with hydroxyurea and the creation of a non-chemical bond.

### 3.4. In Vitro Drug Release

**Figure 8:**
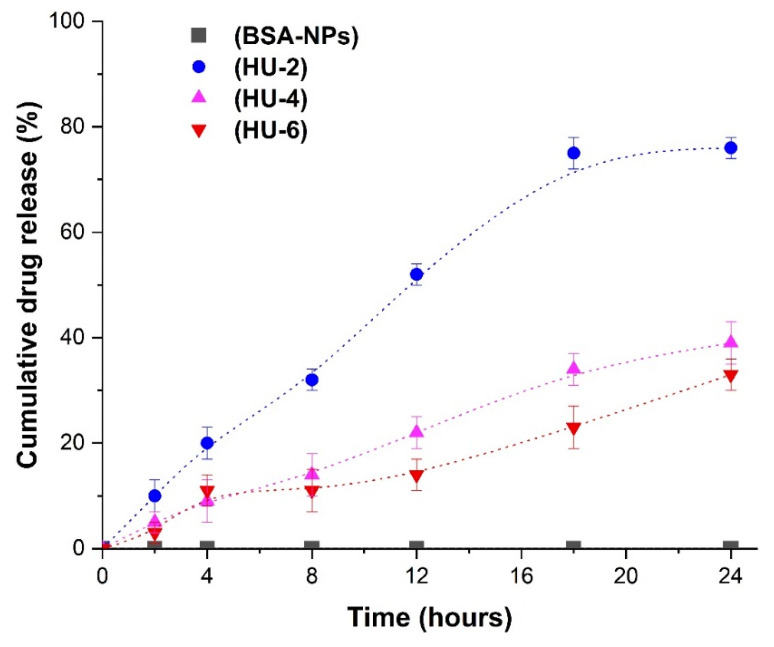
Release of hydroxyurea (HU) from different types of nanoparticles. Data are presented as mean ± SD (n = 3).

HU release profiles from BSA nanoparticles (BSA-HU-NPs) were analyzed for samples obtained by the inclusion method (Figure 8). The samples obtained at the initial concentration of HU 2; 4; 6 mg/mL were marked as HU-2, HU-4, HU-6, respectively. We obtained the yield for samples HU-2, HU-4 and HU-6 = 76 ± 5%, 38 ± 7% and 32 ± 5%, respectively; the drug was released during the 24 h from BSA–HU-NPs, which were prepared at concentrations of hydroxyurea 2, 4, and 6 mg/mL. It should be noted that the release of the HU was significantly slower after period of 24 h for each of the three samples. Approximately 50 ± 5% of hydroxyurea was released from the HU-2 sample within 12 h. Subsequently, the total yield of the drug was 76 ± 5% within a period of 24 h. Two samples with higher drug concentrations (HU-4 and HU-6) showed slow release, which is 38 ± 7% and 32 ± 5% HU within a period of 24 h, respectively. Thus, the data obtained by us can be compared with the results of a study on in vitro release of drugs using the same biologically active substances with human serum albumin nanoparticles [33]. This enables the continuous action on cancer cells, reaching a long-term effect on the viability of cells over time, in contrast to the full release of the drug during the period of 12 h.

## 4. Conclusions

The possibility of obtaining HSA and BSA nanoparticles stabilized by the combined action of urea and cysteine is shown. Differences in the dispersion and stability of albumin particles of various origin are found. When BSA is used for the synthesis of nanoparticles, smaller particles are formed (average particle diameter 162 ± 5 nm), which are more stable (Zeta-Potential 22–27 mV) and uniform (polydispersity index 0.11). These indicators of particles of biopolymers are important in their application for the transport of drugs. It is known that HSA differs from BSA only by one link [44] but differs significantly in conformation. It is possible to add urea to albumin of various origins in different ways changes the conformation of protein molecules and macromolecules respectively formed under this action form particles that differ in characteristics. It was found that the immobilization of nanoparticles of both HSA and BSA with the antitumor drug hydroxyurea occurs successfully with the use of adsorption and inclusion methods. The degree of drug immobilization (Encapsulation Efficiency) is high in all cases, from 62% to 69% for HSA and 68–78% for BSA. In vitro studies of nanoparticles immobilized with an antitumor drug showed that BSA prolonged the release of HU from the polymer matrix. Thus, in some cases, no more than 35% of the drug is released in 24 h, which indicates the possibility of obtaining a prolonged dosage form. The following publications will present the results of biomedical tests of the synthesized samples of NPs immobilized with an antitumor drug.

## Figures and Tables

**Table 1:** Characteristics of HSA and BSA nanoparticles containing absorbed HU.

Type of Initial Albumin	Initial Protein Concentration mg/mL	Particles Size ± SD (nm)		PI ± SD		Zeta-Potential (mV) ±SD		Encapsulation Efficiency (%) ± SD
		Before HU Adsorption	After HU Adsorption	Before HU Adsorption	After HU Adsorption	Before HU Adsorption	After HU Adsorption	
BSA	10	157 ± 2	175 ± 3	0.093 ± 0.010	0.181 ± 0.010	−23 ± 1	−24 ± 1	74 ± 2
	20	163 ± 3	178 ± 5	0.122 ± 0.015	0.199 ± 0.010	−26 ± 1	−27 ± 1	73 ± 2
	30	167 ± 5	183 ± 6	0.202 ± 0.020	0.227 ± 0.010	−25 ± 1	−23 ± 1	70 ± 2
HSA	30	216 ± 9	277 ± 2	0.148 ± 0.015	0.200 ± 0.010	−24 ± 1	−22 ± 0	68 ± 2

**Table 2:** Characteristics of HSA- and BSA-derived nanoparticles containing HU incorporated using inclusion procedure.

Type of Initial Albumin, mg/mL	Particles Size ± SD (nm)	PI ± SD	Zeta-Potential (mV) ± SD	Nanoparticle Yield, Protein %	Encapsulation Efficiency ± SD
BSA (2 mg/mL)	239 ± 10	0.242 ± 0.015	−20 ± 3	100%	78 ± 1
HSA (2 mg/mL)	330 ± 12	0.287 ± 0.010	−17 ± 2	100%	69 ± 2
